# Cellular Senescence in Normal Mammary Gland and Breast Cancer. Implications for Cancer Therapy

**DOI:** 10.3390/genes13060994

**Published:** 2022-06-01

**Authors:** Chaido Sirinian, Stavros Peroukidis, Katharina Kriegsmann, Dimitrios Chaniotis, Angelos Koutras, Mark Kriegsmann, Anastasios D. Papanastasiou

**Affiliations:** 1 Molecular Oncology Laboratory, Department of Medicine, Division of Oncology, University of Patras, 265 04 Patras, Greece; hsirinian@upatras.gr (C.S.); angkoutr@otenet.gr (A.K.); 2 Panarkadikon General Hospital, 221 00 Tripolis, Greece; panio@upatras.gr; 3 Department of Hematology, Oncology and Rheumatology, University Hospital Heidelberg, 69120 Heidelberg, Germany; katharina.kriegsmann@med.uni-heidelberg.de; 4 Department of Biomedical Sciences, University of West Attica, 122 43 Athens, Greece; dchaniotis@uniwa.gr; 5 Institute of Pathology, University of Heidelberg, 69117 Heidelberg, Germany; mark.kriegsmann@med.uni-heidelberg.de

**Keywords:** mammary gland, breast cancer, cellular senescence, chemotherapy, IHC, GL13

## Abstract

Cellular senescence (CS) is a major homeostatic biological process, which plays a key role in normal tissue development and provides protection from stressful cell insults. The role of CS in mammary-gland development and breast cancer is not well understood. While there is a lack of experimental data on the role of CS in the development of the pre-pubertal mammary gland, there is evidence for a biphasic senescence response in adult normal-mammary-epithelial cells, where the bypass of the first senescence barrier (M0) seems to be a key step in the development of premalignant lesions, with genetic abnormalities that resemble in situ breast carcinoma. Further, there is accumulating evidence for the role of cellular senescence in breast-cancer response, regarding treatment and patient outcome. Here, we review the current literature on cellular senescence, in epithelial-mammary cells, breast-cancer cells, and breast-tumor-microenvironment-resident cells. Furthermore, we discuss its putative role in breast-cancer response, regarding treatment and disease progression. In addition, we provide preliminary evidence of CS in breast-cancer-microenvironment cells, such as tumor-associated fibroblasts and tumor-infiltrating lymphocytes, by employing the novel GL13 lipofuscin stain, as a marker of cellular senescence.

## 1. Introduction

Development of the human breast initiates from the milk lines of the fetus’s ventral surface, at the fifth week of gestation [1,2]. Until the 15th week of gestation, the breast bud will undergo the necessary cellular changes, towards mesenchymal condensation, on the chest wall at the site of mammary-gland development [1,3]. The differentiation of basal cells towards myoepithelial cells, an important step with a role in the branching morphogenesis of the gland, takes place between the 23rd and 28th weeks of gestation, and it is characterized by the synthesis of basement-membrane proteins, such as fibronectin, collagen, and laminin [4]. Almost at the same time (weeks 20 to 32), the mesenchyme differentiates into fat within the collagenous stroma, to encompass mammary lobes and their subdivisions. Finally, canalization of cords and branching of the lobuloalveolar glandular structures takes place, during the last two months of gestation [1].

Mammary-gland development and function, in adult life, is largely dependent on steroid hormones [1,5]. After birth, the re-initiation of normal breast development takes place at puberty, coinciding with the onset of the cyclical secretion of the major sex hormones, estrogen and progesterone, which are the driving force of mammary-gland development and function in the adult life. Estrogen acts on the estrogen receptor (ER) of mammary-epithelial cells, inducing proliferation and causing duct elongation and epithelium thickening [2]. At the same time, estrogen affects, in a positive way, the periductal stromal elements, which supports expanding ducts and lobules [5]. On the other hand, progesterone, in conjunction with insulin and growth hormone, are responsible for breast glandular differentiation and the development and growth of the lobuloalveolar units, during puberty [5,6]. The cycling secretion pattern during the menstrual cycle, of estrogen and progesterone, leads to cycles of proliferation, branching, involution, and remodeling of the mammary gland epithelium as well as the related hormone-responsive stroma, during the reproductive years of females and pregnancy [1,5]. These repetitive proliferative cycles in mammary gland function render breast-epithelial and stromal cells subject to constant cellular stress and, thus, prone to DNA damage, leading to the development of replicative stress. Replicative stress is a bona fide mechanism, underlying the generation of genetic lesions (mutations and/or copy number variations), implicated in the initiation of human neoplasia and the progression of premalignant lesions to in situ and invasive carcinomas, when referring to epithelial malignancies [7,8,9,10]. Breast cancer is a highly heterogenous disease, both at the intra- and inter-tumoral levels, but a major subdivision consists of ER-positive and ER-negative tumors that present, with distinct precursor lesions, clinical behavior, and response to therapies [11,12,13].

Multiple regulatory and/or protective mechanisms (apoptosis, anoikis, cellular senescence, autophagy) not only act during normal mammary-gland development but also safeguard cellular integrity, from intrinsic or extrinsic stress factors, which could lead to breast carcinogenesis [14,15,16,17]. These mechanisms play a role, not only in the development of the human mammary gland during gestation and infancy but also in breast function (proliferation, branching, involution, remodeling) during adult life. Deregulation or inhibition of these mechanisms in the human breast can lead to neoplasia [18,19,20,21].

Cellular senescence is a complex molecular and biochemical pathway, with a role both in the regulation of tissue development and function and as an important homeostatic mechanism, acting to preserve cellular/tissue integrity, after multiple stressogenic insults [22,23,24]. The role of cellular senescence in normal mammary-gland development as well as in the initiation and progression of breast carcinoma remains unclear. In this review, we attempt to give an overview of the relevant literature in the field and, at the same time, provide a glimpse into our unpublished preliminary data, concerning cellular senescence in breast cancer.

## 2. Senescence in Normal and Non-Tumorigenic Human Mammary Epithelial Cells

Cellular senescence was first described by Hayflick, as the limited proliferative capacity of primary-fibroblast cells propagated in culture [22]. This phenomenon, termed replicative cellular senescence, is dependent on telomere shortening as primary cells divide, manifested by an irreversible growth arrest and related to cell aging [25,26]. Nevertheless, cellular senescence is a highly complex phenotype, with many intermediate stages, and it seems that replicative senescence, as described by Hayflick, is a small part of the whole biological “picture” [27].

### 2.1. Paradigm of Normal Human Mammary Epithelial Cells (HMECs)

One of the first paradigms, of the cellular-senescence-pathway complexity, is primary mammary epithelial cells, where the basic rule of irreversible growth arrest of senescent cells seems to “bend”, via a population of variant HMECs that escape cellular senescence, through methylation-dependent *CDKN2A* gene silencing [28,29,30]. This process, which possesses evolutionary selection characteristics, leads mammary-epithelial cells into a state of “agonescence” [31]. Agonescence is characterized by stable cell numbers, SA-b-gal (senescence-associated β -galactosidase) staining, and, in contrast to senescence, by BrDU incorporation, cell death, and genomic instability. The intriguing implications of the agonescence state, in mammary neoplastic transformation, is that HMECs at that stage possess chromosomal structural aberrations reminiscent of premalignant and in situ carcinoma lesions in the breast [31,32]. Thus, mammary-epithelial cells that, spontaneously, escape senescence (M0), through p16 protein downregulation, can enter a “transformation” state, in which specific additional genetic or epigenetic hits could lead to premalignant lesions, such as atypical ductal hyperplasia (ADH) and, finally, invasive carcinoma [33]. In this immortalization procedure, through senescence escape/bypass of normal HMEC populations, in addition to the hypermethylation of the p16 promoter, multiple pathways and proteins have been implicated. Prostaglandin cyclooxygenase-2 (COX2) upregulation has been reported in senescence-escaped HMECs, while COX2 expression coincides with areas of *CDKN2A* hypermethylation, in histologically normal human-mammary-gland tissue. COX2 overexpression is, biologically, related to increased prostaglandin synthesis, angiogenesis, and invasion [34]. In addition, p16 (*CDKN2A*) downregulation, through inhibition of proteolytic degradation of p53, in an HMEC-specific manner, stabilizes p53 protein levels, inducing a p53-dependent transcriptional program and a proliferation suppression [35]. The *TP53* gene is a bona fide driver of breast carcinogenesis and is found, mutated, in almost 80% of triple negative (that is hormone-receptor- and HER2-negative tumors) breast cancers [36]. Thus, p16 downregulation could be an early event in mammary-epithelial-cell transformation, whereas p53 inactivation could be a spatiotemporally subsequent hit, leading a premalignant lesion to in situ carcinoma.

Finally, multiple cell-culture models for HMECs have identified critical factors for immortalization and transformation, through senescence bypass. Interestingly, one of the most efficient systems for human mammary-epithelial-cell immortalization is the expression of the human papilloma-virus-related oncogenes, E6 and E7, while tumor suppressor proteins, such as pRb, p53, telomerase, and inhibitors of cyclin-dependent kinases (p21, p27 and p57), have an established role in immortalization and transformation of HMECs in culture [37,38].

### 2.2. The Paradigm of the MCF10A Non-Tumorigenic Mammary Cell Line

A powerful and well-studied cell-culture-model system, recapitulating breast-cancer progression, is the MCF10A cell line. MCF10A is an immortalized mammary epithelial cell line, with the capacity to form spheroids with hollow lumens in Matrigel substrate, mimicking in part, breast-terminal-duct lobular units [39]. Intriguingly, these cells, inherently, lack the *CDKN2A* gene locus, affecting p16 tumor suppressor expression, resembling the methylation-dependent *CDKN2A* downregulation, described for HMECs that escape senescence and introduce cells into the state of “agonescence”, as described by Romanov et al. [31]. At the same time, MCF10A cells, as shown by us and others, are able to evade oncogene-induced senescence (OIS), upon overexpression of a classical oncogene (*HRAS*), indicating that escape of cellular senescence is an early, critical step in the transformation process in the breast [40,41,42].

## 3. Cellular Senescence in Breast Carcinoma

As discussed above, cellular senescence is a barrier to human carcinogenesis, which lies early in the immortalization and transformation processes of human neoplasia [43,44]. Further, cellular senescence presents significant differences between normal human fibroblasts and mammary-epithelial cells, in its course of establishment and escape [45,46]. All the above indicate a highly complex cellular-senescence phenotype, the difficulties to study its presence, and the consequences on the senescent cell and the microenvironment.

An intriguing question is: do human-malignant-epithelial neoplasms encompass senescent cancer cells? Even recent studies indicate that cellular senescence is present in the premalignant state (e.g., lung adenomas) and is absent in malignant lesions [47]; adequate analytical tools are, still, missing to fully address the aforementioned issue. The same question applies, also, for breast cancer, where breast-cancer cell lines, when assessed by the SA-b-gal marker, are negative for the senescence phenotype but can stain positive by either gene manipulations, such as HER2 overexpression and PKCη knock-down, or cancer-therapy-relevant treatments (anti-HER2 or Doxorubicin) [48,49,50,51]. These data indicate that, while breast-cancer cells do not senesce, under specific stress conditions, such as the depletion of an important gene product or treatment with antitumor agents, they enter a cellular senescence state, characterized by positive SA-b-gal staining. Furthermore, when it comes to breast-cancer tissue from human patients, the lack of analytical tools for use with Formalin Fixed Paraffin Embedded (FFPE) tissue makes senescence detection a daunting task [52,53].

**Figure 1:**
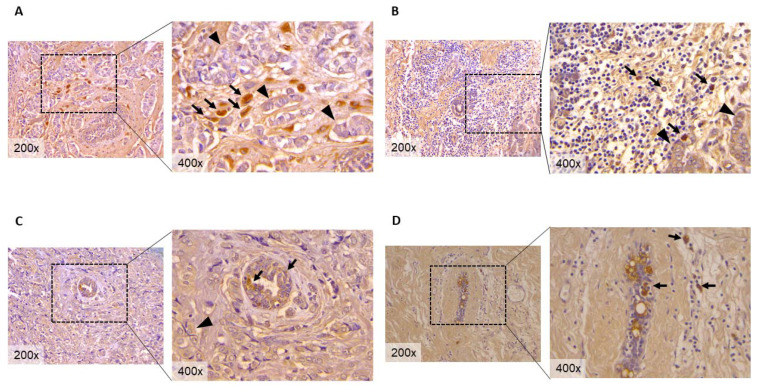
GL13 (SenTraGor™) immunohistochemical stain of tumor-associated fibroblasts (**A**), tumor-infiltrating lymphocytes (**B**), and cancer-entrapped normal-like mammary gland acini (**C**), in breast cancer. A post-treatment specimen (neoadjuvant setting), showing GL13 positive staining in the stroma and mammary remnants (**D**). Arrows indicate GL13 positive staining, while arrowheads depict breast-cancer cells. SenTraGor™ (GL-13) immunohistochemical staining was performed, in accordance with the instructions of the manufacturer and as previously described [55].

In order to identify, in human-breast-cancer-archived tissue, traces of cellular senescence, we employed a novel reagent named GL13 (SenTraGor™), immunohistochemically staining a panel of unselected invasive carcinoma, of no special type (Appendix A) [54,55,56]. These preliminary results indicate that the cancerous tissue component of our FFPE samples stained negative for cellular senescence, as judged by GL13 (SenTraGor™) immunohistochemistry (IHC), in accordance with the notion that cancer cells are not senescent (Figure 1A–C, arrowheads). However, we were able to identify, in the tumor microenvironment, positive staining of epithelial cells, in some normal-appearing cancer-entrapped mammary glands, tumor-associated fibroblasts, or tumor-infiltrating lymphocytes (Figure 1A–C, arrows). These data provide preliminary evidence, for the existence of cellular senescence in the breast-cancer microenvironment, in at least three different cell populations (near-normal epithelia, fibroblasts, lymphocytes) that possess important functions in cancer development and progression [57,58,59,60,61]. The presence and role of these “normal” senescent cells in breast cancer is, still, to be elucidated, as well as the impact on patient clinicopathological parameters, such as recurrence, survival, and response to treatment. Finally, the identification of a senescent microenvironment, in a specific subset of human breast cancers, further expands disease heterogeneity, while the impact of the senescent microenvironment on patient outcome remains to be determined [33,62].

## 4. Cellular Senescence and Breast-Cancer Treatment

Another intriguing question is: can breast-cancer cells enter a state of cellular senescence, after treatment initiation, or, in other words, can breast-cancer therapy, either as chemotherapy or a targeted therapy (e.g., anti-ER or anti-HER2), promote therapy-induced senescence (TIS) [63]? As mentioned above, there is a number of studies that have addressed that question, employing breast-cancer cell lines and either chemotherapy regimens or targeted treatments [48,50,64,65]. Doxorubicin is capable of inducing a senescence-like phenotype in MCF-7 breast-cancer cells, after 3 h of exposure, at 1μM concentration of the chemotherapeutic agent [48,66]. The same cell line (MCF-7) is prone to a senescence-like phenotype, after exposure to retinoic acid, as judged by mRNA profiling and SA-b-gal staining [48,67]. In addition, breast-cancer cells overexpressing HER2/neu (HCC1419, SKBR3), when treated with targeted tyrosine kinase inhibitors of HER2/neu (lapatinib, neratinib, afatinib), showed signs of overt cellular senescence, through SA-b-gal staining [50]. However, considering that most breast-cancer cell lines are derived from metastatic breast-cancer-patient specimens, such as pleural effusions (MCF7, MDA-MB-231, SKBR3), which have been, often, exposed to multiple therapeutic agents (for instance, SKBR3 are derived from a patient that had been treated with radiation, steroids, cytoxan and 5-fluorouracil), implying multiple levels and cycles of genetic selection, one should exercise caution, when interpreting results relevant to the senescence-like phenotype identified in these cell lines.

The only way to test for the existence of senescent breast-cancer cells in humans, after breast-cancer treatment, is the employment of pretreatment and posttreatment samples. in the neoadjuvant setting. Nevertheless, this straight-forward experimental approach has several limitations, such as the need for archived fresh-frozen material, for the employment of SA-b-gal stain, the unstable enzymatic activity of b-galactosidase on fresh frozen material, and the irregular periods from neoadjuvant treatment initiation and completion to surgery (from two to eight months). In an attempt to characterize cellular senescence in human breast cancer, after CAF (Cyclophosphamide, Doxorubicin, 5-Fluorouracil) treatment, te Poele and colleagues [52], employed fresh-frozen breast tissue and stained for SA-b-gal, p53, and p16. They identified SA-b-gal positive staining in 41% of their breast-cancer-patient cohort, after treatment, indicating extensive cellular senescence that co-segregated with low p53 and high p16 protein expression. An interesting finding of that study was that SA-b-gal staining was confined to cancer cells, while the tumor cell microenvironment (fibroblasts, lymphocytes, normal epithelia, etc.) was completely negative, opposite to what one might expect. from normal cells under a constant chemotherapeutic insult.

In order to recapitulate the findings from chemotherapy-induced senescence, in breast-cancer cell lines, we employed a small cohort of breast-cancer patients that received neoadjuvant chemotherapy, for whom we had pre- and post-therapy FFPE tissue availability (*n* = 10). IHC staining with GL13 (SenTraGor™) showed the presence of cellular senescence in normal cells, of some post-therapy samples in the tumor microenvironment, such as fibroblasts and normal-like epithelial cells (Figure 1D, arrows), but not in therapy-escaped tumor cells. In our opinion, a model that could explain these findings is that a population of treated tumor cells, indeed, enter a state of cellular senescence, from which some get cleared (immune surveillance) and some escape or bypass senescence [68,69]; however, in any case, at the time of surgery, when a significant period of time has passed from initiation and completion of treatment, there are no remaining senescent breast-cancer cells.

The occurrence of primary normal senescent cells, as an adverse effect of chemotherapy, has been previously described [70], and these cells can contribute to local and systemic inflammation, increasing chemotherapy-drug toxicity and the fatigue of cancer patients [71,72]. The identification of normal senescent cells, in the tumor microenvironment, might be a novel biomarker for senescence-dependent fatigue induction, in a subpopulation of breast-cancer patients, which could predict therapy toxicity for those patients [73].

## 5. Future Perspectives

Cellular senescence has a role, both in normal tissue/organ development and homeostasis as well as in human neoplastic disease [53,74]. In normal tissue, cellular senescence, on the one hand, regulates aspects of physiological function and, on the other hand, acts as a protective mechanism, against cellular stress and oncogenesis [15]. In malignant neoplasms, cellular senescence can be identified in cancer cells and/or the tumor microenvironment, and the net effect of the senescent phenotype, on patient outcome, remains to be elucidated [63,65,70].

**Figure 2:**
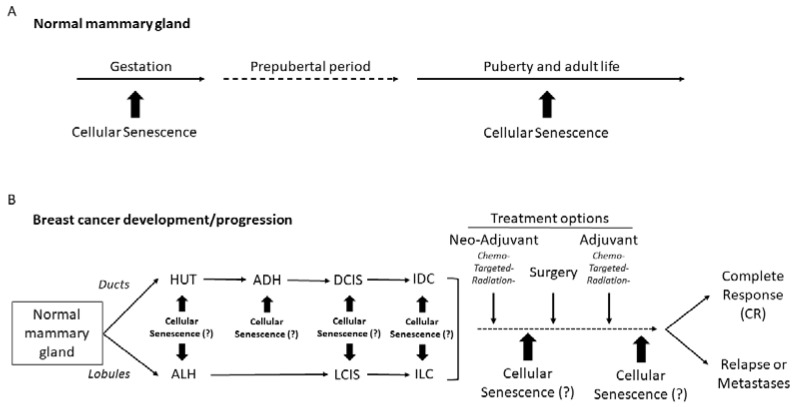
Graphic timeline of events in normal mammary-gland development (**A**) and breast-cancer (IDC and ILC) initiation, progression, and treatment (**B**), depicting the possible stages where cellular senescence may play a significant role in vivo. Question marks (?) indicate stages of possible cellular senescence involvement, with ambiguous effects on carcinogenesis and response to treatment. ADH: atypical ductal hyperplasia; ALH: atypical lobular hyperplasia; DCIS: ductal carcinoma in situ; HUT: hyperplasia of usual type; IDC: invasive ductal carcinoma; ILC: invasive lobular carcinoma; LCIS: lobular carcinoma in situ.

In normal mammary-gland development, the exact function of cellular senescence is not well understood, whilst in the adult breast, it seems to have a protective role against all forms of pro-oncogenic cellular insults [75]. However, while mammary-epithelial cells spontaneously bypass senescence, in the early stages of the transformation process is an obligatory step on the way to malignant transformation, so full-blown invasive breast cancer, most probably, is devoid from the cellular-senescence phenotype, especially at the invasive front [30,33,45]. Nevertheless, cellular senescence in the breast-carcinoma microenvironment might have a significant role in disease development and progression as well as in the response to treatment (Figure 2) [61].

An important aspect, relevant to the cellular-senescent phenotype, is our capability to correctly detect and identify senescent cells [27]. The most frequently used markers are SA-b-gal reactivity, Ki-67 protein absence, high p16 protein expression, and trimethylated histone H3 lysine 9 (H3K9me3) staining [76,77,78,79]. Still, even the combination of different biomarkers cannot, undoubtedly, identify cells as senescent or just in a senescence-like growth-arrest state. Thus, the presence of senescent cells, especially on human FFPE tissue, is hard to prove, and ongoing research points to a combination of markers (e.g., Ki-67, p16, SenTraGor™) that will define a cell as being truly senescent [55,80,81].

The difficulties of senescence-phenotype identification linger, also, for breast cancer, especially in the archived FFPE tissue settings and in routine pathology practice. There are contradicting reports on the existence of cellular senescence, in human epithelial-malignant neoplasms. In breast cancer, te Poele et al. [52] were able to identify, through SA-b-gal staining of archived fresh-frozen tissue, senescent breast-cancer cells in 10% (2 out of 20) of their treated cohort, while our preliminary data (Figure 1A–C) on breast cancer and data from others on lung cancer [47] indicate that senescent carcinoma (epithelial) cells are, probably, extremely rare. Nevertheless, further work and biomarker combination are needed, in order to, fully, characterize the senescent phenotype of breast-cancer cells and of the relevant microenvironment.

The improvement of our capacity to simply, accurately, and cost effectively identify the senescent phenotype in breast-cancer FFPE samples will further expand our understanding on the molecular pathology of breast cancer and provide opportunities for the implementation of a personalized approach to breast-cancer treatment, through the new field of senotherapeutics [62,81].

## Appendix A

Sentragor^TM^ (GL13) staining was done in accordance with the protocol of the manufacturer. In brief, 4 μm thick paraffin sections were mounted on Superfrost Plus microscope slides (BDH Laboratory Supplies, Menzel), and sections were deparaffinized in xylene and rehydrated in descending concentrations of ethanol. Endogenous peroxidase activity was blocked, by immersion in 0.03% H_2_O_2_, for 15 min. Slides were washed with 1xTBS, followed by 5 min incubation in 50% and 70% ethanol. Sentragor was added dropwise on the slides, covered with a coverslip, and left for 8 min at RT. The remaining stain was washed out in 50% EtOH, followed by washes with 0.5% Triton/TBS and 1xTBS. Then, slides were incubated overnight at 4 °C, with anti-biotin (1:300, ab201341, abcam). The next day, the unbound antibody was washed out with 1xTBS, and slides were incubated with a Dako REAL^TM^ EnVision^TM^ Detection System (Dako), for 30 min at RT. The immunoreaction was visualized by the application of 3,3′-diaminobenzidine (DAB). All slides were counterstained with hematoxylin, dehydrated in ascending ethanol concentrations, immersed in xylene, and mounted.
